# *Agra*, arboreal beetles of Neotropical forests: *pusilla* group and *piranha* group systematics and notes on their ways of life (Coleoptera, Carabidae, Lebiini, Agrina)

**DOI:** 10.3897/zookeys.66.684

**Published:** 2010-11-04

**Authors:** Terry L. Erwin

**Affiliations:** Hyper-diversity Group, Department of Entomology, MRC-187, National Museum of Natural History, Smithsonian Institution, Washington, P.O. Box 37012, DC 20013-7012, USA

**Keywords:** Neotropics, Bolivia, Brazil, Ecuador, Perú, rainforest, canopy

## Abstract

Revisions of two new species groups of the genus Agra Fabricius are presented with the following species described as new: *pusilla* group - Agra cruciaria **sp. n.** (Brazil), Agra grace **sp. n.** (Ecuador,Perú), Agra max **sp. n.** (Brazil), Agra minasianus **sp. n.** (Brazil),

Agra notpusilla **sp. n.** (Brazil), Agra pseudopusilla **sp. n.** (Brazil); piranha group - Agra ce **sp. n.** (Perú), Agra risseri **sp. n.** (Bolivia,Brazil), Agra maia **sp. n.** (Bolivia), Agra piranha **sp. n.** (Ecuador); Agra tiputini **sp. n.** (Ecuador). Species of these two groups have adults that are the smallest in the entire genus, although this does not indicate they are closely related based on other attributes. All species are Amazonian in distribution.

## Introduction

The purpose of this 17th contribution in my series of papers with diagnoses of new taxa and redescriptions of known taxa in the beetle genus Agra Fabricius, 1801 (Carabidae) is to present a revision of the species in the *pusilla* and *piranha* groups. For the previous papers, see (Erwin (1978, 1982a, 1982b, 1983, 1984, 1986, 1991, 1993, 1996, 1998, 2000a, 2000b, 2000c, 2002), Erwin and Pogue (1988), and Arndt et al. (2001). New species descriptions provided here will not be the end of the story. New species of this incredibly speciose and diverse genus continue to be discovered each year throughout the Neotropics and subtropical México /Texas and northern Argentina.

These beetles belong to the Tribe Lebiini, Subtribe Agrina, of which the genus Agra Fabricius is by far the largest lineage in number of species. Subtribe Agrina consists of those species formerly included in the Subtribe Calleidina (cf. Lorenz 1998, 2005). More than 2000 neotropical/subtropical species of Agra are known from museum collections; however, only some 592 of these have been described since Fabricius erected the genus in 1801, plus three species described in the very late 1700’s by Olivier and Fabricius, but originally placed in the Linnaean genus Carabus. The sister group was hypothesized to be an African/Madagascan group near Callidiola Jeannel 1949, however Casale (1998) argues against that. His analysis suggested that Agra is more closely aligned with Physoderina, as the adelphotaxon. Certainly, the female stylomere 2 fits that hypothesis in a general way and the male median lobe is not Calleida or Callidiola like. However, there is little in the overall “gestalt" that might lead one to conclude these lineages are at all related. It is time for comparative DNA analysis, I suggest, to determine where these higher lebiine lineages fall out on an updated classificatory scheme. Unfortunately, in the fine contribution of Ober and Maddison (2008), the Physoderina were not included, even though they saw possible monophyly amongst Calleidina (sensu Casale 1998), Agrina, Metallicina etc. This will mean that specimens of the *pusilla* group need to be in the DNA mix of samples because of their clearly, based on structural attributes, less derived features amongst all the other species groups of this very diverse genus. And, members of Physoderina will need to be in the mix, as well.

The *pusilla* group contains what appear to be the least derived lineage of Agra and obtaining DNA from them will be crucial in locating the sister lineage – an additional reason for this present revision is making the species and their localities more broadly available, so that fresh specimens can be appropriately acquired and identified. The ***piranha*** group apparently is more derived than the ***pusilla*** group, but both groups are composed in part of little blue beetles and I thought it better to treat them together for purposes of easier identification and classification.

Likely, adult Agra are predaceous on other arthropods; one specimen of another species group dissected had fragments of termites in its gut contents. Adults also have been observed drinking exudates from young new shoots and young leaves on a variety of tree species, as well as feeding on pollen (Arndt et al. 2001). Adults are active on tree surfaces in the canopy and along forest edges at night, as well as in suspended dry leaves in the understory; in addition, at least one species group is often found on savannah grasses. Adult tarsi are adapted for running on the surfaces of leaves with pads similar to those found in adult Chrysomelidae and Cerambycidae. Adults rest under these leaves “concealed" with legs and antennae tucked close to the body, the beetle aligned perfectly with the midrib of the leaf; in the case of grass-dwelling species they align with the culms. Agra adults are nocturnal and commonly fly to lights at night and into Malaise traps, however, none are known so far from ground level flight-intercept traps. These beetles have a potent defensive secretion from paired glands in their abdomen that, in field tests, bats definitely do not like (Erwin 1978). And, males of many species of this genus have special ventral patches of setae or pubescence that suggest they may waft pheromones to attract females. If correct, these chemicals have not been explored for their composition or possible uses. Known larvae are thought to occur normally under bark of standing trees, probably in burrows of other insects, and are thought to be predatory (Arndt et al. 2001). A few larvae have been obtained by rearing from eggs of known females and with insecticidal fogging techniques before dawn (Arndt et al. 2001), the latter indicating the larvae may wander on the tree surfaces at night.

## Specimens and methods

Methods and species concepts follow those previously described (Erwin and Kavanaugh 1981; Kavanaugh and Erwin 1991). The species validation and diagnosis format follows as closely as possible that suggested in Erwin and Johnson (2000) and as used in Erwin (2000a, 2004). Measurements of length (ABL, SBL) and width (TW) follow those of Ball (1972) and Kavanaugh (1979): ABL (apparent body length), measured from apex of labrum to apex of longer elytron; SBL (standardized body length), equals the sum of the lengths of the head (measured from apex of clypeus to a point on midline at level of the posterior edge of compound eyes), pronotum (measured from apical to basal margin along midline), and elytron length (measured from apex of scutellum to apex of the longer elytron); and TW, (total width), measured across both elytra at their widest point.

Included in this study are a total of 22 specimens from the National Museum of Natural History, Washington, DC (NMNH); H. Perrin, J. Menier, Museu National d’Historie Naturelle, Paris (MNHNP ); A. Kuska, Institut Zoologique, Warsaw, Poland (WAR); S. Fragoso, M. Monné, Brazil National Collection, Museu Nacional, Rio de Janeiro, Brazil (BNCRio); M. Baccis, British Museum (Natural History), London, England (BMNH); O.V. Ferreira and J. Jurberg, Ozwaldo Cruz Institute, Rio de Janeiro, Brazil, (ZIKAN); R. Woodruff, Florida Department of Agriculture, Gainesville (FSCA). Note that the curators named were on duty at the time of the loan; many of these have since retired.

The habitus images of the adult beetles portray most of the character states referred to in the keys provided. Illustrations of male genitalia are standard for descriptive taxonomy of carabid beetles. The habitus images of the adults were made with a Visionary Digital^TM^ high resolution imaging system. Figure captions include an ADP number, which is a unique identification number for the specimen that was illustrated or imaged and links the specimen and associated illustrations and/or image to additional information in electronic databases at the NMNH. All scale lines are 0.5mm.

Geographical data are presented for species based on all known specimens available at the time of manuscript preparation. Georeferences have been determined from locality information provided on specimen labels; only those exact Georeferences that are provided on the label are placed in quotes, otherwise I have estimated the Georeferences as closely as possible from places, mileage, etc. listed on the label and searched with Google Earth Pro. Latitude and longitude are reported in decimal degrees. Distribution maps are provided for the species (Figs 10, 11). Here, English vernacular names are proposed, as common names are becoming increasingly needed in conservation and/or agricultural and forestry applications.

The species list below, as well as arrangement of descriptions that follows is ordered alphabetically.

## Accounts of taxa

### Genus Agra

Fabricius, 1801

Agra  Fabricius, 1801:224. Type-species: Agra aeneaFabricius 1801:224, named first among three species described by Fabricius. Designated by Erwin (1982a). Agridia Chaudoir, 1861:109. Type-species: Agridia platyscelis Chaudoir (1861: 109), named first among two species described by Chaudoir. Designated by Erwin (1982a).

#### Diagnostic combination.

Elegant Canopy Beetles

During evolution toward a canopy domain and away from a likely under canopy sister group, Agra adults acquired numerous generic-level autapotypic features as follows: head elongate with prognathate mandibles, securiform labial ultimate palpomeres (Erwin 1982a), extended cranium, and constricted neck; prothorax elongate and tubular, plural sutures effaced; tarsomeres (Erwin 1982a) dilated with setiferous pads beneath, claws explanate and pectinate; elytron with latero-basal sinus and latero-apical callus, apex medially and laterally toothed or somewhat produced, apical margin truncate, sinuate, or medially lobed; and male venter variously adorned with setal or pubescent patches; female reproductive system adapted to egg-laying deep in substrate (telescopic) (Erwin 2002) with (usually) stout, apically-armed with ensiform setae, stylomere 2. Defense system very large (Erwin 1982). Size. ABL=6.0mm to 29.0mm; TW=1.5mm to 6.0mm.

#### Note.

For descriptions of species groups previously recognized, refer to (Erwin (1996, 1998, 2002).

### The pusilla species-group

Members of the *pusilla* group are the smallest of the genus Agra and in part, of a beautiful blue color and matte luster with highly contrasting bicolored femora.

#### Diagnosis:

Femur bicolored. Back of head rounded and sparsely punctate. Adult males with extensive ventral tarsomere pads on both front and middle legs. Prothorax markedly punctate. Elytral interneurs of uni- and/or biseriate rows of cribriform punctures. Aedeagus with narrow spatulate apex. Female stylomere 2 short and arcuate, glabrous, and apically armed with two ensiform setae, as in Fig. 7.

#### Note:

The known composite range of the *pusilla* group extends from Amazonian Ecuador and Perú across southeastern Brazil and into the Mata Atlântica.

#### Included Species

Agra cruciaria Erwin, sp. n. 	Brazil

Agra grace Erwin, sp. n. 	Ecuador, Perú

Agra max Erwin, sp. n. 	Brazil

Agra minasianus Erwin, sp. n.	Brazil

Agra notpusilla Erwin, sp. n.	Brazil

Agra perforata Liebke, 1938 	Brazil

Agra pseudopusilla Erwin, sp. n. 	Brazil

Agra pusilla Chaudoir, 1847	Brazil

#### Key to the species of the pusilla group of  Agra Fabricius, 1801

**Table:** 

1	Elytra bright metallic blue	2
1’	Elytra dull matte smoky black or smoky black-blue, not bright	3
2(1)	Elytral interneurs each with a single row of cribriform punctures	Agra grace, sp. n.
2’	Elytral interneurs each with double rows of cribriform punctures at least in part in apical half	Agra max, sp. n.
3(1’)	Antennal scape unicolorous testaceous, or testaceous with slight dorso-apical infuscation	4
3’	Antennal scape bicolored, ventrally testaceous, dorsally infuscated	6
4(3)	Head posteriorly and pronotum with surface brassy	Agra cruciaria, sp. n.
4’	Head and pronotum matte smoky black , shiny or not	5
5(4’)	Elytra constricted at apical third, side margin moderately arcuate, apex moderately lobed medially	Agra notpusilla, sp. n.
5’	Elytra not constricted at apical third, side margin straight, apex markedly lobed medially	Agra pusilla Chaudoir
6(3’)	Elytral apex shallowly lobed medially, lobe rounded	Agra pseudopusilla, sp. n.
6’	Elytral apex moderately lobed medially, lobe obtusely dentate	7
7(6’)	Frons laterad, anterior to eye markedly rugose; larger species (ABL = 8.2 mm)	Agra perforata Liebke
7’	Frons laterad, anterior to eye, unicarinate and smooth, smaller species (ABL = 7.2 mm)	Agra minasianus, sp. n.

#### Agra cruciaria

Erwin sp. n.

urn:lsid:zoobank.org:act:7906629D-6846-4348-A3CD-B7A3031C7AE9

Figs 3 10 

##### Holotype:

**Brazil**: Rio de Janeiro, Rio de Janeiro, Corcovado, 585m, 22.9517°S, 43.2116°W, 5 May 1958 (C.A. Campos Seabra & M. Alvarenga)(NMNH: ADP 070044, male).

##### Derivation of specific epithet.

The epithet “*cruciaria*" is a Latin adjective meaning “of/pertaining to the cross/torture" and is based upon the large cross on Corcovado flooded each night with high powered lights that attract insects by the millions and is the type locality of this species.

##### Proposed English vernacular name.

Cross Elegant Canopy Beetle.

##### Diagnosis.

With the attributes of the genus and species-group as described above and medium sized for the *pusilla* group. Adults with black integument; head behind eyes and prothorax with slight brassy reflections. Frons laterad unicarinate and rugose. Occiput with sparse punctures, some punctures with short setae.

Elytra with moderately lobed apex.

##### Description.

(Fig. 3). *Size*: Small, ABL = 6.4 – 7.7 mm, SBL = 5.75 – 6.48 mm, TW = 1.74 –1.8 mm. *Color:* Head black with brassy reflection posteriorly, pronotum with brassy reflections; antennae blackish blue with scape testaceous and with slight metallic blue reflections , mouthparts piceous, and legs and tarsi blackish blue, with mostly testaceous femur. *Luster:* Head, pronotum and legs shiny metallic, elytra matte smoky-blue. *Head:* Labrum moderately elongate and truncate apically, barely emarginate medially. Frons medially raised and smooth, laterally depressed and unicarinate and rugose. Gena with hind angles broadly rounded to constricted neck in males. Genae and occiput moderately densely and coarsely some punctures setiferous.

*Prothorax:* Slightly broader medially, flared basally; surface with dense punctures, many setiferous; lateral elongate callous with single row of setiferous punctures along middle. *Pterothorax:* Elytron markedly convex, intervals slightly costate, interneurs of rows of somewhat irregularly shaped punctures that are double in some places, apex slightly oblique and moderately lobate, apical dentation asymmetric, lateral tooth small, acute, sutural apex slightly produced, narrowly pointed. Metasternum sparsely setiferous in male. *Legs:* Normal in male. *Abdomen*: Abdominal sterna III to VII of male moderately and bilaterally setiferous; sternum VII of male barely emarginated, corners rounded. *Male genitalia:* Phallus (Fig. 3) elongate and narrow with ostium elongate, nearly half the length of the phallus, apex a narrowly lobate expansion of distal end, this slipper-shaped in lateral aspect. Parameres small, left twice the size of the right, both broadly rounded. *Female ovipositor:* Female unknown.

##### Dispersal potential.

These beetles are macropterous and are probably capable of flight; they are swift and agile runners.

##### Way of life.

Adults of other Agra species are found in the canopy of rainforest trees; larvae of this genus are found under the bark of these trees, however they must also roam on the surface, as they have been collected by insecticidal fogging techniques in the very early morning before first light. Members of Agra cruciaria occur at midland altitudes in the Mata Atlântica. Adults are active in May, the rainy season.

##### Other specimens examined.

**Brazil**: Minas Gerais, (MNHNP: ADP 060040, male paratype).

##### Geographic distribution.

(Fig. 10). This species is currently known from the type locality and an unknown location in the State of Minas Gerais.

##### Notes.

Right antenna glued to card of holotype not of this specimen.

#### Agra grace

Erwin sp. n.

urn:lsid:zoobank.org:act:2B0E0A90-4A13-404A-9047-8D61E61E7419

Figs 1 4 10 

##### Holotype:

**Perú**: Madre de Dios, Pakitza, Tachigali Trail /47, 324m, “11.9352°S, 71.3039°W," 6 October 1991 (T.L. Erwin & M.G. Pogue)(NMNH: BIOLAT 012952, female).

##### Derivation of specific epithet.

The epithet “*grace*" is an eponym, based on the given name of the Peruvian Ornithologist, Grace Servat, who has shared the bird-infested Amazon and Andes with me for many years, including two of the known localities of this species, including the type locality.

##### Proposed English vernacular name.

Grace’s Elegant Canopy Beetle.

##### Diagnosis.

With the attributes of the genus and species-group as described above and small sized for the *pusilla* group. Adults with blue integument; head behind eyes and prothorax with metallic reflections. Frons laterad unicarinate and very finely rugose. Occiput not punctate, some fine rugae present. Elytra with moderately lobed apex in female, more so in male. Hind coxae of male multisetiferous.

**Figure 1.**
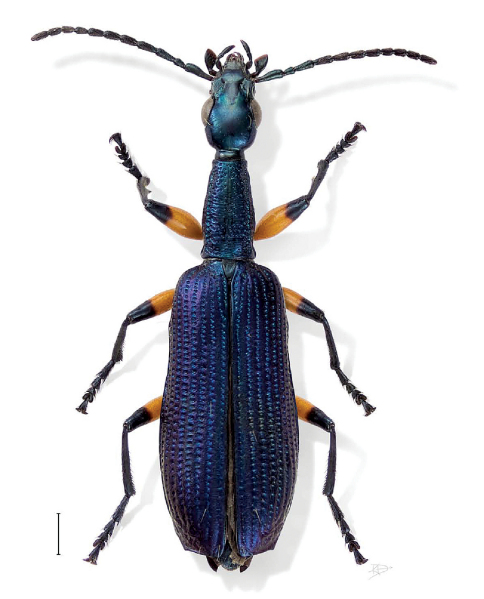
Agra grace Erwin, sp. n., dorsal aspect (BIOLAT 012952).

##### Description.

(Figs 1, 4). *Size*: Very small, ABL = 5.71 – 6.1 mm, SBL = 4.77 – 6.67 mm, TW = 1.44 – 1.66 mm. *Color:* Head and pronotum bright blue, body metallic blue; antennae and mouthparts piceous, scape and antennomeres 2 and 3 with slight metallic blue reflections. *Luster:* Shiny metallic, elytra somewhat matte. *Head:* (Fig. 1) Labrum moderately elongate and truncate apically. Frons medially raised and smooth, laterally depressed, unicarinate, not rugose. Gena slightly tapered with broadly rounded corners to constricted neck in both sexes. Occiput not punctate, some fine rugae present. *Prothorax:* Slightly broader medially, flared basally; surface with dense coarse punctures, some setiferous; lateral elongate callous with single row of punctures, along middle. *Pterothorax:* Elytron (Fig. 1) markedly convex, intervals slightly costate, interneurs of rows of somewhat laterally ovate punctures, apex truncate, barely oblique, apical dentation asymmetric, lateral tooth broad, obtuse, sutural apex not produced. Metasternum sparsely setiferous in both sexes. *Legs:* Normal. *Abdomen*: Abdominal sterna III to VII of both sexes moderately and bilaterally setiferous; sternum VII of both sexes barely emarginate, corners rounded. *Male genitalia:* Phallus (Fig. 4) elongate and narrow with ostium not elongate, extended to about 1/3 the length of phallus, apex a small rounded lobe. Parameres small, left twice the size of the right, both broadly rounded. *Female ovipositor:* Stylomere 2 as in *Agra notpusilla* (Fig. 7).

##### Dispersal potential.

These beetles are macropterous and are probably capable of flight; they are swift and agile runners.

##### Way of life.

Adults are found in the canopy of rainforest trees; known larvae of this genus (Arndt et al. 2001) are found under the bark of these trees, however they must also roam on the surface, as they have been collected by insecticidal fogging techniques in the very early morning before first light. Members of Agra grace occur at lowland altitudes in the Amazon Basin. Adults are active in September — October, the transition season between dry and wet seasons. The holotype was fogged from a medium-sized tree with lianas and suspended dead leaves; the area fogged was from 2 meters up to 15 meters in the tree. The Ecuadorian paratype was fogged from a mixed canopy consisting of the trees Pseudolmedia laevis (Ruiz & Pav.) J.F. Macbr., Protium cf. nodulosum, and Eschweilera cf. coriacea. The Peruvian paratype was fogged from a species of the tree genus Pouteria.

##### Other specimens examined.

**Ecuador:** Orellana, 1 km S Onkone Gare Camp, Entomology Transect, 216m, “0.6569°S, 76.4527°W," 7 October 1994 (T.L. Erwin, et al.)(NMNH: ADP 087438, male paratype). **Perú**: Madre de Dios, Pakitza, 324m, “11.9352°S, 71.3039°W," 9 September 1988 (T.L. Erwin & B.D. Farrell)(NMNH: BIOLAT 008430, male paratype).

##### Geographic distribution.

(Fig. 10). This species is currently known from Perú and Ecuador.

##### Notes.

Males are smaller than females.

#### Agra max

Erwin sp. n.

urn:lsid:zoobank.org:act:20642068-3310-4BCC-B9A8-69D9FBEABB41

Fig. 10 

##### Holotype:

**Brazil:** Santa Catarina, Nova Teutonia, 823m, 27.047°S, 52.394°W, 4 February 1938 (F. Plaumann)(WAR: ADP 004374, female).

##### Derivation of specific epithet.

The epithet “*max*" is an eponym, based on the given name of Max Liebke, an early pioneer in the taxonomy of the genus Agra.

##### Proposed English vernacular name.

Max’s Elegant Canopy Beetle.

##### Diagnosis.

With the attributes of the genus and species-group as described above and frons laterally unicarinate and rugose; occiput finely punctate; all elytral interneurs in apical half with double rows of cribriform punctures.

##### Description.

*Size*: Small, ABL = 7.0 mm, SBL = 5.95 mm, TW = 1.5 mm. *Color:* Head and pronotum black with bluish reflections, venter metallic blue, elytra matte blue, antennae and mouthparts piceous with slightly bluish reflections, scape rufous with shiny infuscated apex. *Luster:* Shiny metallic, elytra matte metallic. *Head:* Labrum moderately elongate and truncate apically. Frons medially raised and smooth, laterally depressed, slightly rugose. Gena slightly markedly rounded to constricted neck in female. Genae and occiput with sparse coarse punctures, some setiferous.

*Prothorax:* Short, slightly broader medially, flared basally; surface with dense and coarse punctures, some setiferous; lateral elongate callous with single row of setiferous puncture along middle. *Pterothorax:* Elytron markedly convex, intervals slightly costate, interneurs of rows of somewhat laterally ovate punctures, doubled in some places, apex moderately oblique and lobed, apical dentation asymmetric, lateral tooth small, broad, obtuse, sutural apex not produced. Metasternum sparsely setiferous in females. *Legs:* Normal in female. *Abdomen*: Abdominal sterna III to VII of female moderately and bilaterally setiferous; sternum VII of female barely emarginate, corners rounded. *Male genitalia:* Unknown. *Female ovipositor:* Stylomere 2 as in Agra notpusilla (Fig. 7).

##### Dispersal potential.

These beetles are macropterous and are probably capable of flight; they are swift and agile runners.

##### Way of life.

Adults of other Agra species are found in the canopy of rainforest trees; known larvae of this genus (Arndt et al. 2001) are found under the bark of these trees, however they must also roam on the surface, as they have been collected by insecticidal fogging techniques in the very early morning before first light. Members of Agra max occur at midland altitudes in the Mata Atlântica. Adults are active in February, the dry season.

##### Other specimens examined.

None.

##### Geographic distribution.

(Fig. 10). This species is currently known only from the type locality.

#### Agra minasianus

Erwin sp. n.

urn:lsid:zoobank.org:act:B3072B22-36A7-415D-A2B6-E6FE05270DC0

Fig. 10 

##### Holotype:

**Brazil:** Minas Gerais, (Laferte)(MNHNP: ADP 060090, female).

##### Derivation of specific epithet.

The epithet “*minasianus*" is a Latinized adjective meaning “derived from, or pertaining to" Minas Gerais, a State in Brazil, and the type area.

##### Proposed English vernacular name.

Minas Elegant Canopy Beetle.

##### Diagnosis.

With the attributes of the genus and species-group as described above and scape and legs bicolored, frons laterad, anterior to eye, unicarinate and smooth, prothorax moderately setiferous both laterally and ventrally, and elytra barely constricted at apical third, side margin slightly arcuate, apex moderately lobed medially, lobe obtusely dentate, smaller species.

##### Description.

*Size*: Very small, ABL = 7.2 mm, SBL = 6.24 mm, TW = 1.5 mm. *Color:* Head and prothorax black, legs and scape bicolored; flagellar antennomeres and mouthparts piceous. *Luster:* Matte. *Head:* Labrum moderately elongate and truncate apically, anterior margin slightly emarginate medially. Frons laterad, anterior to eye, unicarinate and smooth. Gena rounded to constricted neck in females. Genae and occiput with sparse coarse punctures, some setiferous.

*Prothorax:* Slightly broader at basal third, constricted near base and flared basally; surface densely punctate, laterally and ventrally setiferous, pronotum apparently with six lateral setae as in other species of the group, but not present on holotype. *Pterothorax:* Elytron markedly convex, intervals not costate, interneurs of rows of somewhat laterally ovate punctures, some in doubles, apex lobate, lobe well developed obtuse projection, apical dentation asymmetric, lateral and sutural apices slightly produced, each obtuse. Metasternum sparsely setiferous in female. *Legs:* Normal. *Abdomen*: Abdominal sterna III to VII of female moderately and bilaterally setiferous; sternum VII of female barely emarginate, corners rounded. *Male genitalia:* Unknown. *Female ovipositor:* Stylomere 2 as in Agra notpusilla (Fig. 7).

##### Dispersal potential.

These beetles are macropterous and are probably capable of flight; they are swift and agile runners.

##### Way of life.

Adults of other Agra species are found in the canopy of rainforest trees; known larvae of this genus (Arndt et al. 2001) are found under the bark of these trees, however they must also roam on the surface, as they have been collected by insecticidal fogging techniques in the very early morning before first light. The single specimen of Agra minasianus has no associated data on its labels.

##### Other specimens examined.

None.

##### Geographic distribution.

(Fig. 10). This species is currently known from Minas Gerais, Brazil.

#### Agra notpusilla

Erwin sp. n.

urn:lsid:zoobank.org:act:988FCC3E-1A4F-439D-833C-66B524B7269F

Figs 7 10 

##### Holotype:

**Brazil:** (MNHNP: ADP 058647, male).

##### Derivation of specific epithet.

The epithet “*pusilla*" is a Latin adjective meaning very little, small, pretty. Although this species resembles Agra pusilla it is “not" that species.

##### Proposed English vernacular name.

Brazilian Elegant Canopy Beetle.

##### Diagnosis.

With the attributes of the genus and species-group as described above and frons laterally multicarinate; elytra with interneurs composed of double rows (in part) of coarse irregularly shaped punctures, apex moderately lobed medially.

##### Description.

(Fig. 7). *Size*: Very small, ABL = 7.74 mm, SBL = 6.58 mm, TW = 1.88 mm. *Color:* Piceous, antennae and legs bicolored, scape and basal 5/6th of femur testaceous; antennal flagellum and mouthparts piceous. *Luster:* Head and abdomen shiny, elytral disc somewhat matte. *Head:* Labrum moderately elongate and truncate apically, slightly emarginate medially. Frons medially raised and smooth, laterally depressed, multicarinate. Gena rounded in female to constricted neck. Genae and with occiput sparse and moderately coarse punctures, some of which likely setiferous in undamaged specimens (no setae present in holotype), and with two larger punctures,. *Prothorax:* Slightly broader in basal third, constricted, and flared basally; surface densely punctate, laterally and ventrally setiferous. *Pterothorax:* Elytron markedly convex, intervals not costate, interneurs of double rows (in part) of somewhat irregularly shaped punctures, apex truncate, moderately lobate, apical dentation asymmetric, lateral tooth small, acute, sutural apex slightly produced, rounded. Metasternum sparsely setiferous in both females. *Legs:* Legs normal. *Abdomen*: Abdominal sterna III to VII of female moderately and bilaterally setiferous; sternum VII of females barely emarginate, corners rounded. *Male genitalia:* Unknown. *Female ovipositor:* Stylomere 2 (Fig. 7).

##### Way of life.

Adults of other Agra species are found in the canopy of rainforest trees; known larvae of this genus (Arndt et al. 2001) are found under the bark of these trees, however they must also roam on the surface, as they have been collected by insecticidal fogging techniques in the very early morning before first light. Members of Agra notpusilla have no recorded information.

##### Other specimens examined.

None.

##### Geographic distribution.

(Fig. 10). This species is currently known from Brazil.

#### Agra perforata

Liebke, 1938

Fig. 10

Agra perforata Liebke 1938: 60.

##### Holotype:

**Brazil:** Rio de Janeiro, Rio de Janeiro, 585m, 22.9517°S, 43.2116°W (WAR: ADP 060035, female).

##### Derivation of specific epithet.

The epithet “*perforata*" is a Latin adjective referring to the cribriform punctures of the elytral interneurs.

##### Proposed English vernacular name.

Perforated Elegant Canopy Beetle.

##### Diagnosis.

With the attributes of the genus and species-group as described above and frons laterally multicarinate; occiput coarsely punctate; all elytral interneurs throughout with double rows of cribriform punctures.

##### Description.

*Size*: Small, ABL = 8.22 mm, SBL = 6.58 mm, TW = 1.88 mm. *Color:* All black with mostly testaceous femur. *Luster:* Head slightly shiny, pronotum and elytra matte black. *Head:* Labrum moderately elongate and rounded apically. Frons medially raised and smooth, laterally depressed, moderately rugose. Gena markedly rounded to constricted neck in female. Genae and occiput with sparse and coarse punctures, some setiferous. *Prothorax:* Short, slightly broader medially, flared basally; surface with dense and coarse punctures, some setiferous; lateral elongate callous with single row of setiferous punctures along middle. *Pterothorax:* Elytron markedly convex, intervals slightly costate, interneurs of double rows of somewhat irregularly-shaped punctures, apex moderately oblique and lobed, apical dentation asymmetric, lateral tooth small, broad, obtuse, sutural apex not produced. Metasternum sparsely setiferous in females. *Legs:* Normal in female. *Abdomen*: Abdominal sterna III to VII of female moderately and bilaterally setiferous; sternum VII of female barely emarginate, corners rounded. *Male genitalia:* Unknown. *Female ovipositor:* Stylomere 2 as in Agra notpusilla (Fig. 7).

##### Dispersal potential.

These beetles are macropterous and are probably capable of flight; they are swift and agile runners.

##### Way of life.

Adults of other Agra species are found in the canopy of rainforest trees; known larvae of this genus (Arndt et al. 2001) are found under the bark of these trees, however they must also roam on the surface, as they have been collected by insecticidal fogging techniques in the very early morning before first light. Members of Agra perforata occur at midland altitudes in the Mata Atlântica.

##### Other specimens examined.

None.

##### Geographic distribution.

(Fig. 10). This species is currently known only from the type locality.

##### Notes.

Additional character state information can be found in Liebke 1938: 60.

#### Agra pseudopusilla

Erwin sp. n.

urn:lsid:zoobank.org:act:B39D7377-B0B7-43D8-AE76-0F3F0EF58ABB

Figs 5 10 

##### Holotype:

**Brazil:** (Comte G. de Mniszech)(MNHNP: ADP 060088, female).

##### Derivation of specific epithet.

The epithet “*pseudopusilla*" refers to the similarity between adults of this species and those of Agra pusilla, treated below.

##### Proposed English vernacular name.

Mniszech’s Elegant Canopy Beetle.

##### Diagnosis.

With the attributes of the genus and species-group as described above and frons laterally multicarinate; occiput coarsely bi-punctate, with several smaller punctures; elytral interneurs with mostly uni-serial rows of cribriform punctures, doubled apico-laterally.

##### Description.

(Fig. 5). *Size*: Small, ABL = 6.57 – 8.63 mm, SBL = 5.67 – 7.15 mm, TW = 1.34 – 2.08 mm. *Color:* Head and pronotum black, elytra smoky black, legs bicolored, antennae and mouthparts piceous, scape with testaceous venter, piceous dorsum. *Luster:* Shiny forebody, matte elytra. *Head:* Labrum moderately elongate and truncate apically, anterior corners rounded. Frons medially raised and smooth, laterally depressed, multicarinate. Gena slightly tapered with broadly rounded corners to constricted neck in both male. Occiput coarsely bi-punctate, with several smaller punctures. *Prothorax:* Slightly broader medially, flared basally; surface with dense and coarse punctures, some setiferous; lateral elongate callous with single row of setigerous punctures along middle. *Pterothorax:* Elytron markedly convex, intervals slightly costate, interneurs of rows of somewhat laterally ovate punctures, doubled in some places, apex oblique, slightly lobed at middle, apical dentation asymmetric, lateral tooth short, acute, sutural apex not produced. Metasternum sparsely setiferous in males. *Legs:* Normal. *Abdomen*: Abdominal sterna III to VII of male moderately and bilaterally setiferous; sternum VII of males barely emarginate, corners rounded. *Male genitalia:* Phallus (Fig. 5) elongate and narrow with ostium not elongate, extended to about 1/2 the length of phallus, apex a small rounded lobe. Parameres small, left twice the size of the right, both moderately rounded. *Female ovipositor:* Female unknown

##### Dispersal potential.

These beetles are macropterous and are probably capable of flight; they are swift and agile runners.

##### Way of life.

Adults of other Agra species are found in the canopy of rainforest trees; known larvae of this genus (Arndt et al. 2001) are found under the bark of these trees, however they must also roam on the surface, as they have been collected by insecticidal fogging techniques in the very early morning before first light. Members of Agra pseudopusilla are labeled Brazil without further information.

##### Other specimens examined.

**Brazil:** (Ehrenreiche)(BNCRio: ADP 070045, male paratype).

##### Geographic distribution.

(Fig. 10). This species is currently known only from Brazil, without specific location.

#### Agra pusilla

Chaudoir, 1847

Figs 6 10

Agra pusilla Chaudoir 1847: 110.

##### Holotype:

**Brazil:** (MNHNP: ADP 060087, male).

##### Derivation of specific epithet.

The epithet “*pusilla*" is a Latin adjective meaning very little, small, pretty.

##### Proposed English vernacular name.

Small Elegant Canopy Beetle.

##### Diagnosis.

With the attributes of the genus and species-group as described above and frons laterally unicarinate and rugose; elytra with interneurs composed of a single row of coarse irregularly shaped punctures, apex markedly lobed medially.

##### Description.

(Fig. 6). *Size*: Very small, ABL = 6.28 – 6.45 mm, SBL = 5.27 – 5.37 mm, TW = 1.32 – 1.46 mm. *Color:* Piceous, antennae and legs bicolored, scape and basal 5/6th of femur testaceous; antennal flagellum and mouthparts piceous. *Luster:* Shiny, elytral disc somewhat matte. *Head:* Labrum moderately elongate and rounded apically, slightly emarginate medially. Frons medially raised and smooth, laterally depressed, unicarinate, and shallowly rugose. Gena slightly tapered-rounded in both sexes to constricted neck. Genae and occiput moderately finely punctate and wrinkled, and with two larger punctures each of which is setiferous. *Prothorax:* Slightly broader medially, flared basally; surface with densely punctures, some setiferous; lateral elongate callous with single row of setiferous punctures along middle. *Pterothorax:* Elytron markedly convex, intervals not costate, interneurs of rows of single somewhat irregularly shaped punctures, apex truncate, markedly lobate, apical dentation asymmetric, lateral tooth small, acute, sutural apex slightly produced, rounded. Metasternum sparsely setiferous in both sexes. *Legs:* Legs normal. *Abdomen*: Abdominal sterna III to VII of male moderately and bilaterally setiferous; sternum VII of males barely emarginate, corners rounded. *Male genitalia:* Phallus (Fig. 6) with ostium elongate, about half the length of the phallus, apex a small rounded lobe with basal corners. Parameres small, left twice the size of the right, both moderately rounded. *Female ovipositor:* Stylomere 2 as in Agra notpusilla (Fig. 7).

##### Dispersal potential.

These beetles are macropterous and are probably capable of flight; they are swift and agile runners.

##### Way of life.

Adults of other Agra species are found in the canopy of rainforest trees; known larvae of this genus (Arndt et al. 2001) are found under the bark of these trees, however they must also roam on the surface, as they have been collected by insecticidal fogging techniques in the very early morning before first light. Members of Agra pusilla occur at lowland altitudes in the Mata Atlântica. Adults are active in October, the rainy season.

##### Other specimens examined.

**Brazil:** Espirito Santo, nr. Itapemirim, Rio Itapemirim, 3m, 21.005°S, 40.834°W, 15 October 1906 (J.F. Zikan)(ZIKAN: ADP 070043, male).

##### Geographic distribution.

(Fig. 10). This species is currently known from eastern Brazil.

##### Notes.

Because of the severe deforestation over the last 100 years in the area where this species was found, it is likely it is now extinct or at least with a much smaller range.

### The piranha species-group

Members of this group are of very small size for the genus and range in color from midnight metallic blue to smoky-black with a somewhat matte luster. Male adults have a much reduced version of the expansive ventral tarsomere pads found in all other species in the genus. Femur unicolored. Occiput and prothorax markedly punctuate. Elytral interneurs of uniseriate rows of cribriform punctures. Aedeagus with typical arrowhead shape. Female stylomere 2 short and arcuate, setiferous, and armed with two ensiform setae. Female stylomere as in Fig. 9.

#### Notes:

The known composite range of the *piranha* group extends from Amazonian Ecuador to Bolivia across into south-central Brazil (Goiás).

#### Included Species

Agra ce Erwin, sp. n. 	Perú

Agra risseri Erwin, sp. n. 	Bolivia, Brazil

Agra maia Erwin, sp. n. 	Bolivia

Agra piranha Erwin, sp. n. 	Ecuador

Agra tiputini Erwin, sp. n. 	Ecuador

#### Key to the species of the piranha group of Agra Fabricius, 1801

**Table:** 

1	Prothorax markedly setiferous both laterally and ventrally, pronotum with 3 long setae on each side	2
1’	Prothorax not setiferous laterally, pronotum with 4 long setae on each side	4
2(1)	Head very broad across occiput, wider than pronotum at its widest	Agra risseri, sp. n.
2’	Head not broad across occiput, narrower than pronotum at its widest	3
3(2’)	Elytra constricted at apical third, side margin markedly arcuate	Agra maia, sp. n.
3’	Elytra barely constricted at apical third, side margin slightly arcuate	Agra ce, sp. n.
4(1’)	Elytra constricted at apical third, side margin markedly arcuate; head markedly narrowed basally	Agra tiputini, sp. n.
4’	Elytra barely constricted at apical third, side margin slightly arcuate; head slightly tapered yet broad basally	Agra piranha, sp. n.

#### Agra ce

Erwin sp. n.

urn:lsid:zoobank.org:act:6A25E44D-7927-4FD0-9FD2-9B37E12458D7

Fig. 11 

##### Holotype:

**Perú:** Madre de Dios, 30 air km SW Puerto Maldonado, 205m, “12.8368°S, 69.2933°W," 10 September 1984 (T.L. Erwin, et al.)(NMNH: ADP 093837, female).

##### Derivation of specific epithet.

The epithet “*ce*" is a combination of pronounceable letters that when joined with the last three letters of the genus name, Agra, spells “grace," for the Peruvian Ornithologist, Grace Servat, who has shared the lowland Amazon and the high Andes with me for many years, including the known localities of this species.

##### Proposed English vernacular name.

Graceful Elegant Canopy Beetle.

##### Diagnosis.

With the attributes of the genus and species-group as described above and elytra and prothorax metallic blue, legs unicolored, frons laterad slightly rugose, prothorax markedly setiferous both laterally and ventrally, and elytra barely constricted at apical third, side margin slightly arcuate.

##### Description.

*Size*: Small, ABL = 8.04 – 9.21 mm, SBL = 7.09 – 8.07 mm, TW = 2.36 – 2.78 mm. *Color:* Head black with bluish reflection posteriorly, body and legs metallic blue; antennae and mouthparts piceous, scape with slight metallic blue reflections. *Luster:* Shiny metallic. *Head:* Labrum moderately elongate and rounded apically. Frons medially raised and smooth, laterally depressed and rugose. Gena almost squared to constricted neck in females. Genae and occiput moderately densely punctate, each puncture setiferous. *Prothorax:* Slightly broader medially, flared basally; surface with dense and coarse setiferous punctures; lateral elongate callous with single row of setiferous punctures along middle. *Pterothorax:* Elytron markedly convex, intervals moderately costate, interneurs of rows of somewhat laterally ovate punctures, apex truncate, barely lobate, apical dentation asymmetric, lateral tooth small, acute, sutural apex slightly produced, narrowly pointed. Metasternum sparsely setiferous in female. *Legs:* Normal in female. *Abdomen*: Abdominal sterna III to VII of female moderately and bilaterally setiferous; sternum VII of female barely emarginated, corners rounded. *Male genitalia:* Unknown. *Female ovipositor:* Stylomere 2 as in Agra piranha (Fig. 9).

##### Dispersal potential.

These beetles are macropterous and are capable of flight; they are swift and agile runners.

##### Way of life.

Adults of other Agra species are found in the canopy of rainforest trees; larvae of this genus are found under the bark of the these trees, however they must also roam on the surface, as they have been collected by insecticidal fogging techniques in the very early morning before first light. Members of Agra ce occur at lowland altitudes in the Amazon Basin. Adults are active in September, the late dry season. The holotype was collected in an Erwin Plot at the type locality; the forest of this plot is designated as a Swamp Forest with internal drainage (Erwin 1985) and is dominated by the palm Mauritia flexuosa L. and the hardwood tree Lueheopsis hoehnei Burret. The holotype was fogged from the later named species. The paratype was attracted to MV light.

##### Other specimens examined.

**Perú**: Madre de Dios, Pakitza, Trocha Uno /14, 324m, “11.9352°S, 71.3039°W," 8 September 1989 (R.A. Faitoute, et al.)(NMNH: BIOLAT 017465, female paratype).

##### Geographic distribution.

(Fig. 11). This species is currently known only from two localities in southeastern Perú.

#### Agra maia

Erwin sp. n.

urn:lsid:zoobank.org:act:4CBBDF28-0E25-4818-A2DA-72D2CE6B3697

Fig. 11 

##### Holotype:

**Bolivia:** Santa Cruz, 4–6 km SSE Buena Vista, Hotel Flora & Fauna, 400–500m, “17.479°S, 63.631°W," 1–10 November 2002 (S.W. Lingafelter)(NMNH: ADP 116043, female).

##### Derivation of specific epithet.

The epithet “*maia*" is a Latinized genitive eponym, based on the given name of Maia Samuel, Executive Producer of the Smithsonian Spotlight program, *The Bug House*, in recognition of the hours of dedication she put into developing the program.

##### Proposed English vernacular name.

Maia’s Elegant Canopy Beetle.

##### Diagnosis.

With the attributes of the genus and species-group as described above and prothorax brassy black, legs unicolored, frons laterad unicarinate, smooth, prothorax markedly setiferous both laterally and ventrally, and elytra markedly constricted at apical third, side margin markedly arcuate.

##### Description.

*Size*: Small, ABL = 8.57 mm, SBL = 7.25 mm, TW = 2.2 mm. *Color:* Head black with faint bluish reflection posteriorly, pronotum brassy black legs and venter black, elytra metallic blue; antennae and mouthparts piceous. *Luster:* Shiny, pronotum brassy and elytra shiny metallic. *Head:* Labrum moderately elongate and moderately rounded apically. Frons medially raised and smooth, laterally depressed and smooth. Gena almost squared to constricted neck in females. Genae and occiput with moderately dense disc each side with four long setae, punctures, most setiferous. *Prothorax:* Slightly broader medially, flared basally; surface densely punctuate, disc each side with four long setae; lateral elongate callous with single row of setiferous punctures along middle. *Pterothorax:* Elytron moderately convex, broadly flared at apical third, intervals moderately costate, interneurs of rows of somewhat laterally ovate punctures, apex truncate, barely lobate, apical dentation asymmetric, lateral tooth small, acute, sutural apex slightly produced, narrowly pointed. Metasternum sparsely setiferous in female. *Legs:* Normal in female. *Abdomen*: Abdominal sterna III to VII of female moderately and bilaterally setiferous; sternum VII of female barely emarginated, corners rounded. *Male genitalia:* Unknown. *Female ovipositor:* Stylomere 2 as in Agra piranha (Fig. 9).

##### Dispersal potential.

These beetles are macropterous and are probably capable of flight; they are swift and agile runners.

##### Way of life.

Adults of other Agra species are found in the canopy of rainforest trees; known larvae of this genus (Arndt et al. 2001) are found under the bark of these trees, however they must also roam on the surface, as they have been collected by insecticidal fogging techniques in the very early morning before first light. Members of Agra maia occur at lowland altitudes in the Amazon Basin. Adults are active in November, the rainy season.

##### Other specimens examined.

None.

##### Geographic distribution.

(Fig. 11). This species is currently known only from the type locality.

#### Agra piranha

Erwin sp. n.

urn:lsid:zoobank.org:act:0B2DB88C-89F6-485D-B2FB-C2B54813D0DC

Figs 8, 9 11 

##### Holotype:

**Ecuador:** Orellana, 1 km S Onkone Gare Camp, Entomology Transect, 216m, “0.6569°S, 76.4527°W," 2 July 1995 (T.L. Erwin, et al.)(NMNH: ADP 087440, male).

##### Derivation of specific epithet.

The epithet “*piranha*" or piraña, is a translation of the Huaorani word, Onkone Gare, the name of the camp near which the holotype was discovered.

##### Proposed English vernacular name.

Piraña Elegant Canopy Beetle.

##### Diagnosis.

With the attributes of the genus and species-group as described above and brassy pronotum, legs unicolored, frons laterad unicarinate, smooth, prothorax not setiferous laterally, and elytra barely constricted at apical third, side margin slightly arcuate, apex truncate, barely lobed medially.

##### Description.

(Fig. 8, 9). *Size*: Very small, ABL = 7.18 – 7.29 mm, SBL = 5.98 – 6.13 mm, TW = 1.76 – 1.86 mm. *Color:* Head black with bluish reflection posteriorly, body and legs with metallic blue reflections, elytra metallic cobalt blue; antennae and mouthparts piceous, scape with slight metallic blue reflections. *Luster:* Shiny metallic, elytra matte. *Head:* Labrum moderately elongate and rounded at corners, slightly emarginate medially. Frons medially raised and smooth, laterally depressed and uni-carinate. Gena slightly tapered, hind angle obtuse to constricted neck in both sexes. Genae and occiput with moderately dense and coarse punctures, some setiferous. *Prothorax:* Slightly broader medially, flared basally; surface densely punctuate, disc each side with three long setae; lateral elongate callous with single row of non-setiferous punctures along middle. *Pterothorax:* Elytron markedly convex, intervals moderately costate, interneurs of rows of somewhat laterally ovate punctures, apex truncate, barely lobate, apical dentation asymmetric, lateral tooth small, acute, sutural apex not produced, rounded. Metasternum sparsely setiferous in both sexes. *Legs:* Middle tibia of male with dense brush of short setae on medial margin. *Abdomen*: Abdominal sterna III, IV, and V of both sexes sparsely setiferous bilaterally; sternum VII of both sexes very slightly emarginate. *Male genitalia:* Phallus (Fig. 8) elongate, narrow, with moderately broad arrow-shaped apex; ostium elongate. Parameres small, left twice the size of the right, both broadly rounded. *Female ovipositor:* Stylomere 2 (Fig. 9).

##### Dispersal potential.

These beetles are macropterous and are probably capable of flight; they are swift and agile runners.

##### Way of life.

Adults are found in the canopy of terre firme rainforest trees; known larvae of this genus (Arndt et al. 2001) are found under the bark of these trees, however they must also roam on the surface, as they have been collected by insecticidal fogging techniques in the very early morning before first light. Members of Agra piranha occur at lowland altitudes in the Amazon Basin. Adults are active in July and October, in both the rainy and transition seasons. The holotype was fogged from the hardwood Eschweilera cf. laevicarpa in the familyLecythidaceae. The paratype was fogged from a mixed canopy consisting of the palms Iriartea deltoidea Ruiz. & Pav. and Wettinia maynensis Spruce, and the hardwood Macrolobium cf. ischnocalx, plus an unidentified species ofApocynaceae.

##### Other specimens examined.

**Ecuador:** Rio Tiputini, Erwin Transect, 232m, “0.63173°S, 76.14420°W," 23 October 1998 (T.L. Erwin, et al.)(NMNH: ADP 117227, female paratype).

##### Geographic distribution.

(Fig. 11). This species is currently known from the Ecuadorian Amazon Basin.

#### Agra risseri

Erwin sp. n.

urn:lsid:zoobank.org:act:358EFA50-1C15-4476-946C-0E35E74FB47C

Figs 2 11 

##### Holotype:

**Bolivia:** Santa Cruz, 4–6 km SSE Buena Vista, Hotel Flora & Fauna, 383m, “17.479°S, 63.631°W," 27–29 October 2000 (J.E. Wappes & R. Morris)(FSCA: ADP 115786, female).

##### Derivation of specific epithet.

The epithet “*risseri*" is a Latinized genitive eponym, based on the surname of Dr. Paul G. Risser, outgoing Chairman of the National Board of the Smithsonian’s National Museum of Natural History, in honoring his long and invaluable service to the Smithsonian Institution.

##### Proposed English vernacular name.

Risser’s Elegant Canopy Beetle.

##### Diagnosis.

With the attributes of the genus and species-group as described above and legs unicolored, head very broad across occiput, wider than pronotum at its widest,frons laterad unicarinate, smooth, prothorax markedly setiferous both laterally and ventrally, and elytra slightly constricted at apical third, side margin barely arcuate.

**Figure 2.**
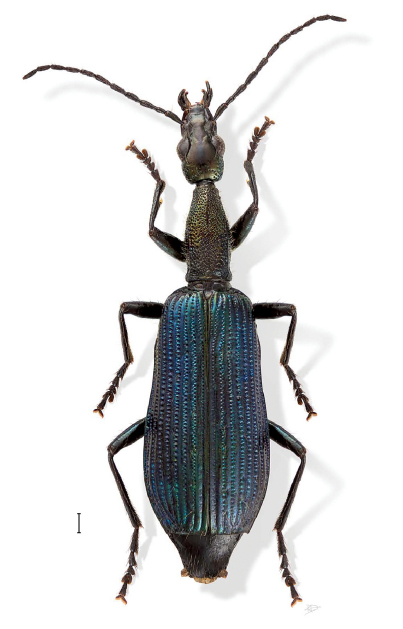
Agra risseri Erwin, sp. n., dorsal aspect (ADP 115786).

**Figures 3–6.**
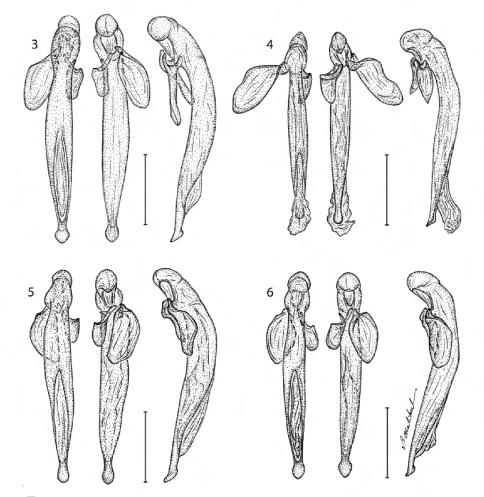
3 Agra cruciaria Erwin, sp. n., male genitalia (dorsal, ventral, left lateral aspects) (ADP 070044) **4** Agra grace Erwin, sp. n., male genitalia (dorsal, ventral, left lateral aspects) (ADP 087638) **5** Agra pseuopusilla Erwin, sp. n., male genitalia (dorsal, ventral, left lateral aspects) (ADP 070045) **6** Agra pusilla Chaudoir, sp. n., male genitalia (dorsal, ventral, left lateral aspects) (ADP 060087).

**Figure 7.**
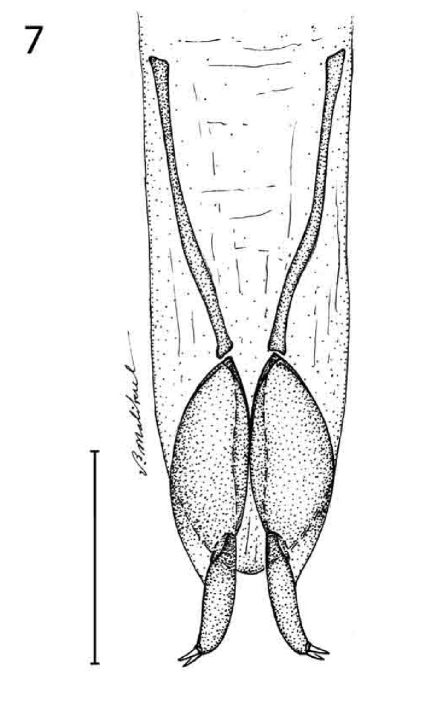
Agra notpusilla Erwin, sp. n., female stylomeres (dorsal aspect) (ADP 058647).

**Figures 8–9.**
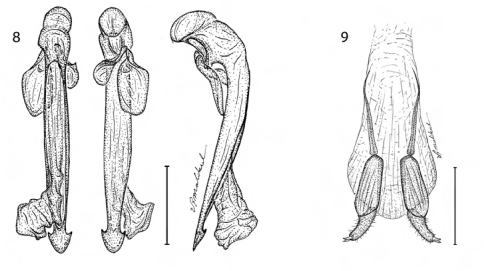
8 Agra piranha Erwin, sp. n., male genitalia (dorsal, ventral, left lateral aspects) (ADP 087440) **9** Agra piranha Erwin, sp. n., female stylomeres (dorsal aspect) (ADP 117227).

##### Description.

(Fig. 2). *Size*: Small, ABL = 9.42 – 10.45 mm, SBL = 8.14 – 8.77 mm, TW = 2.4 – 3.08 mm. *Color:* Head, body, and appendages black, pronotum with brassy reflection elytra metallic blue. *Luster:* Shiny. *Head:* (Fig. 2) Labrum moderately elongate and slightly rounded at anterior corners. Frons medially raised and smooth, laterally depressed and unicarinate, smooth. Gena almost squared to constricted neck in females. Genae and occiput with moderately dense punctures, most setiferous. *Prothorax:* Slightly broader medially, flared basally; surface with dense punctures, some setiferous; lateral elongate callous with single row of setiferous punctures along middle. *Pterothorax:* Elytron (Fig. 2) with discal area flat, intervals moderately costate, interneurs of rows of somewhat laterally ovate punctures, apex truncate, barely lobate, apical dentation asymmetric, lateral tooth small, acute, sutural apex slightly produced, narrowly pointed. Metasternum sparsely setiferous in female. *Legs:* Normal in female. *Abdomen*: Abdominal sterna III to VII of female moderately and bilaterally setiferous; sternum VII of female shallowly emarginate, corners rounded. *Male genitalia:* Unknown. *Female ovipositor:* Stylomere 2 as in Agra piranha (Fig. 9).

##### Dispersal potential.

These beetles are macropterous and are probably capable of flight; they are swift and agile runners.

##### Way of life.

Adults of other Agra species are found in the canopy of rainforest trees; known larvae of this genus (Arndt et al. 2001) are found under the bark of these trees, however they must also roam on the surface, as they have been collected by insecticidal fogging techniques in the very early morning before first light. Members of Agra risseri occur at lowland to midland altitudes in the Amazon Basin. Adults are active in October, the rainy season.

##### Other specimens examined.

**Brazil:** Goiás, Jataí (Jatahy), 736m, 17.880°S, 51.720°W (BMNH: ADP 004375, female paratype).

##### Geographic distribution.

(Fig. 11). This species is currently known from Bolivia and Brazil.

**Figure 10.**
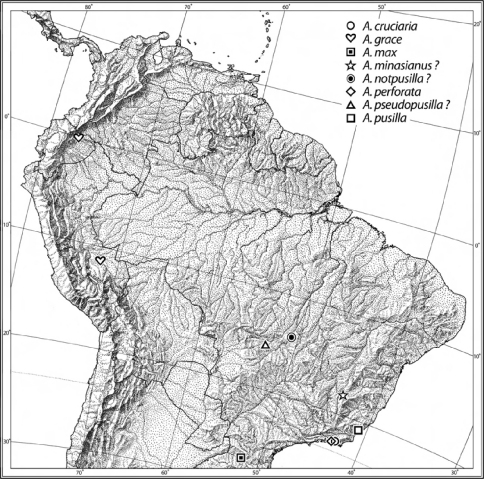
Distribution map of the species of the pusilla group. Names marked with a “?" do not have precise localities on the specimen label(s).

**Figure 11.**
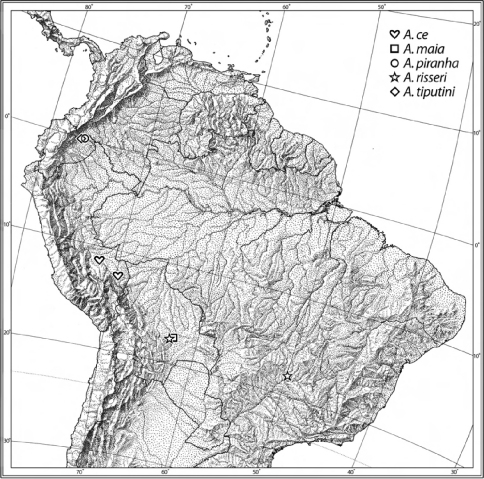
Distribution map of the species of the piranha group.

#### Agra tiputini

Erwin sp. n.

urn:lsid:zoobank.org:act:97A92342-25C2-461C-95F1-52FDDB63741B

Fig. 11 

##### Holotype:

**Ecuador**: **Ecuador:** Orellana, Tiputini Biodiversity Station, Rio Tiputini, Erwin Transect, 232m, “0.63173°S, 76.14420°W," 23 October 1998 (T.L. Erwin, et al.)(NMNH: 117233, female).

##### Derivation of specific epithet.

The epithet “*tiputini*" is the name of the Research Station and the river near which the holotype was collected.

##### Proposed English vernacular name.

Tiputini Elegant Canopy Beetle.

##### Diagnosis.

With the attributes of the genus and species-group as described above and pronotum brassy, legs unicolorous, lateral depression of frons unicarinate, smooth, gena and occiput with sparse and moderately small setigerous punctures plus two larger ones; elytra constricted at basal third, flared at apical third, side markedly arcuate, intervals not costate.

##### Description.

*Size*: Small, ABL = 5.98 – 7.17 mm, SBL = 5.98 – 6.04 mm, TW = 2.04 mm. *Color:* Head black with bluish reflection posteriorly, body and legs with metallic blue reflections, elytra metallic cobalt blue; antennae and mouthparts piceous, scape with slight metallic blue reflections. *Luster:* Shiny metallic, elytra matte. *Head:* Labrum moderately elongate and rounded at corners, slightly emarginate medially. Frons medially raised and smooth, laterally depressed and smooth. Gena slightly tapered, hind angle obtuse to constricted neck in both female. Genae and occiput with sparse setiferous punctures, some coarsely so. *Prothorax:* Slightly broader medially, slightly flared basally; surface with dense punctures, disc each side with four long setae, with short setae both basally and apically; lateral elongate callous with single row of non-setiferous puncture along middle. *Pterothorax:* Elytron moderately convex, flared at apical third, intervals not costate, interneurs of rows of somewhat laterally ovate cribriform punctures, apex truncate, barely lobate, apical dentation asymmetric, lateral tooth small, acute, sutural apex not produced, rounded. Metasternum sparsely setiferous in females. *Legs:* Legs normal. *Abdomen*: Abdominal sterna III, IV, and V of females sparsely setiferous bilaterally; sternum VII of female very slightly emarginate. *Male genitalia:* Unknown. *Female ovipositor:* Stylomere 2 as in Agra piranha (Fig. 9).

##### Dispersal potential.

These beetles are macropterous and are probably capable of flight; they are swift and agile runners.

##### Way of life.

Adults are found in the canopy of terre firme rainforest trees; known larvae of this genus (Arndt et al. 2001) are found under the bark of these trees, however they must also roam on the surface, as they have been collected by insecticidal fogging techniques in the very early morning before first light. Members of Agra tiputini occur at lowland altitudes in the Amazon Basin. Adults are active in February and October, the dry season and the transition season. The holotype was fogged from a mixed canopy consisting of the crowns of the palms Iriartea deltoidea Ruiz. & Pav. and Wettinia maynensis Spruce, and the hardwood Macrolobium cf. ischnocalyx. The paratype was fogged from the hardwood family Myrtaceae. The February specimen is very teneral suggesting that the dry season triggers pupation and emergence.

##### Other specimens examined.

Orellana, 1 km S Onkone Gare Camp, Entomology Transect, 216m, “0.6569°S, 76.4527°W," 8 February 1995 (T.L. Erwin, et al.)(NMNH: ADP 087439, male paratype).

##### Geographic distribution.

(Fig. 11). This species is currently known from Amazonian Ecuador.

## Discussion

The relatively narrow tarsi, spatulate apex of the median lobe of the male genitalia, and very small size of its adults suggest that the *pusilla* species group represents the most basal lineage in the evolution of the hyper diverse genus Agra, given that more highly derived states of tarsal width, male genitalic form, and size are found in the *platyscelis*, *famula*, and *formicaria* species groups, as well as the *piranhna* group, treated herein? In the “Introduction" above, I suggested that fresh specimens should be collected to provide DNA that can be sequenced and analyzed concomitant with those of other adult Agrina genera from Africa to determine the adelphotaxon. Subsequent to such an analysis, a better understanding of the evolution of structural attributes among Agra lineages and species will be possible than it is at present.

## Supplementary Material

XML Treatment for Agra

XML Treatment for Agra cruciaria

XML Treatment for Agra grace

XML Treatment for Agra max

XML Treatment for Agra minasianus

XML Treatment for Agra notpusilla

XML Treatment for Agra perforata

XML Treatment for Agra pseudopusilla

XML Treatment for Agra pusilla

XML Treatment for Agra ce

XML Treatment for Agra maia

XML Treatment for Agra piranha

XML Treatment for Agra risseri

XML Treatment for Agra tiputini
